# Iron Modulates Butyrate Production by a Child Gut Microbiota *In Vitro*

**DOI:** 10.1128/mBio.01453-15

**Published:** 2015-11-17

**Authors:** Alexandra Dostal, Christophe Lacroix, Lea Bircher, Van Thanh Pham, Rainer Follador, Michael Bruce Zimmermann, Christophe Chassard

**Affiliations:** a Institute of Food, Nutrition and Health, Laboratory of Food Biotechnology, ETH Zurich, Switzerland; b Microsynth AG, Next Generation Sequencing Group, Switzerland; c Institute of Food, Nutrition and Health, Laboratory of Human Nutrition, ETH Zurich, Switzerland; University of Michigan Medical School

## Abstract

The aim of this study was to investigate the effect of iron (Fe) availability on butyrate production in the complex bacterial ecosystem of the human gut. Hence, different Fe availabilities were mimicked in an *in vitro* colonic fermentation model (the polyfermenter intestinal model called PolyFermS) inoculated with immobilized gut microbiota from a child and in batch cultures of the butyrate producer *Roseburia intestinalis*. Shifts in the microbial community (16S rRNA sequencing and quantitative PCR), metabolic activity (high-performance liquid chromatography), and expression of genes involved in butyrate production were assessed. In the PolyFermS, moderate Fe deficiency resulted in a 1.4-fold increase in butyrate production and a 5-fold increase in butyryl-coenzyme A (CoA):acetate CoA-transferase gene expression, while very strong Fe deficiency significantly decreased butyrate concentrations and butyrate-producing bacteria compared with the results under normal Fe conditions. Batch cultures of *R. intestinalis* grown in a low-Fe environment preferentially produced lactate and had reduced butyrate and hydrogen production, in parallel with upregulation of the lactate dehydrogenase gene and downregulation of the pyruvate:ferredoxin-oxidoreductase gene. In contrast, under high-Fe conditions, *R. intestinalis* cultures showed enhanced butyrate and hydrogen production, along with increased expression of the corresponding genes, compared with the results under normal-Fe conditions. Our data reveal the strong regulatory effect of Fe on gut microbiota butyrate producers and on the concentrations of butyrate, which contributes to the maintenance of host gut health.

## IMPORTANCE

Fe deficiency is one of the most common nutritional deficiencies worldwide and can be corrected by Fe supplementation. In this *in vitro* study, we show that environmental Fe concentrations in a continuous gut fermentation model closely mimicking a child’s gut microbiota strongly affect the composition of the gut microbiome and its metabolic activity, particularly butyrate production. The differential expression of genes involved in the butyrate production pathway under different Fe conditions and the enzyme cofactor role of Fe explain the observed modulation of butyrate production. Our data reveal that the level of dietary Fe reaching the colon affects the microbiome, and its essential function of providing the host with beneficial butyrate.

## INTRODUCTION

Iron (Fe) is an essential element for almost all living organisms, including most bacteria, and is involved in many biological processes, such as respiration, H_2_ production, and DNA biosynthesis (1, 2). It is well known that Fe not only acts as a cofactor in many enzymatic processes but also regulates gene expression in bacteria, such as virulence genes (3) or genes involved in metabolic pathways (4).

The human gut is an environment where bacteria may encounter a broad range of different Fe concentrations. The gut microbiota can use Fe sources from the diet, and dietary levels of Fe can vary widely, from small amounts of poorly bioavailable nonheme Fe in plant-based diets to high concentrations of bioavailable Fe given as oral Fe supplements to treat Fe deficiency (5, 6). Few studies so far have investigated the effect of Fe on the microbial ecosystem of the human gut, considering the high prevalence of Fe deficiency worldwide and its treatment with high-dose Fe supplements (5). Studies in humans and animals all reported changes in microbial composition due to Fe supplementation, mostly increases in *Enterobacteriaceae* (7, 8) and *Bacteroides* spp. and decreases in bifidobacteria and lactobacilli (7, 9–13), but the studies lacked any investigation of the production of gut microbial metabolites. Using a combination of *in vivo* and *in vitro* models and human trials, we recently showed strong effects of Fe supplementation and low-Fe conditions on the microbial ecosystem of the gut and, also, on the production of the short-chain fatty acids (SCFA) acetate, propionate, and butyrate (14–17). In rats, Fe deficiency of both the diet and the host resulted in a marked decrease in propionate and butyrate production, while *Bacteroides* spp. and *Roseburia* spp./*Eubacterium rectale* decreased and lactobacilli and *Enterobacteriaceae* increased (14, 16). Subsequent Fe supplementation partially restored the microbial composition, promoted gut microbiota metabolic activity, and in particular, increased butyrate production. Similar findings were obtained using *in vitro* colonic fermentation models with immobilized child gut microbiota operated under different Fe conditions (15). The chelation of Fe by 2,2′-dipyridyl led to a strong decrease in butyrate and propionate production, while acetate and the intermediate products lactate and formate accumulated in fermentation effluents, along with a decrease in butyrate-producing *Roseburia* spp./*E. rectale* and propionate-producing *Bacteroides* spp. (15).

Our *in vivo* and *in vitro* experiments show that Fe modulates the gut microbiota metabolic activity and, hence, one of the main contributions of the gut microbiota to host health (18, 19). Butyrate in particular has had beneficial properties attributed to it, since it is the main energy source for colonocytes and is involved in cellular apoptosis and NF-κB signaling and, thus, has anticancer and anti-inflammatory effects (20, 21). Moreover, the degradation of indigestible fibers from the diet by the gut microbiota and the resulting metabolites can contribute an additional 10% of daily dietary energy to the host (18).

The mechanisms by which Fe influences butyrate production of the gut microbiota are not clear. Therefore, the aim of the present study was to confirm the already observed effects of Fe availability on the gut microbiota using a different fecal donor (15) and to further investigate the underlying mechanisms by which Fe deficiency and Fe supplementation influence the complex bacterial community of the human gut and its metabolic function, particularly butyrate production. For this study, we used a new design of the polyfermenter intestinal model called PolyFermS, which is a model of colonic *in vitro* continuous fermentation inoculated with immobilized gut microbiota from a child to investigate the effects of differing Fe concentrations and bioavailabilities in the fermentation medium set to mimic the chyme entering the colon. The differing Fe conditions were achieved by the addition of the chelator 2,2′-dipyridyl and by ferrous sulfate supplementation. This new design of the PolyFermS fermentation model allows accurate parallel testing of different factors in each fermentation reactor and comparison to the results from a control reactor, all of which are seeded with the same microbiota (22, 23). This represents a major improvement from our previous study (15), where tests were performed sequentially. This model allows highly controlled studies of gut bacterial communities without confounding factors of the host or differences in diet, as are found *in vivo* (15, 24).

Importantly, we also analyzed the effect of Fe on the model butyrate producer *Roseburia intestinalis*, due to the high abundance of *Roseburia* spp. in the human gut microbiota (2 to 15% of total bacteria) and its contribution to butyrate production (25). Already in our previous study (15), we found that *Roseburia* spp. were among the bacterial groups most influenced by Fe availability. Shifts in the microbial ecosystem due to differing Fe levels were monitored by 16S rRNA sequencing and quantitative PCR (qPCR). The metabolic functioning of the gut microbiota, as well as that of the butyrate producer *R. intestinalis*, was interpreted by using predictive metagenomic analysis of prevalent functional pathways and by measuring metabolite production, as well as the expression of key genes in the butyrate production pathway, such as the butyryl-coenzyme A (CoA):acetate CoA-transferase gene *butCoAT* (25–28).

## RESULTS

### Gut microbiota compositions in fermentation reactors with different Fe concentrations.

**FIG 1:**
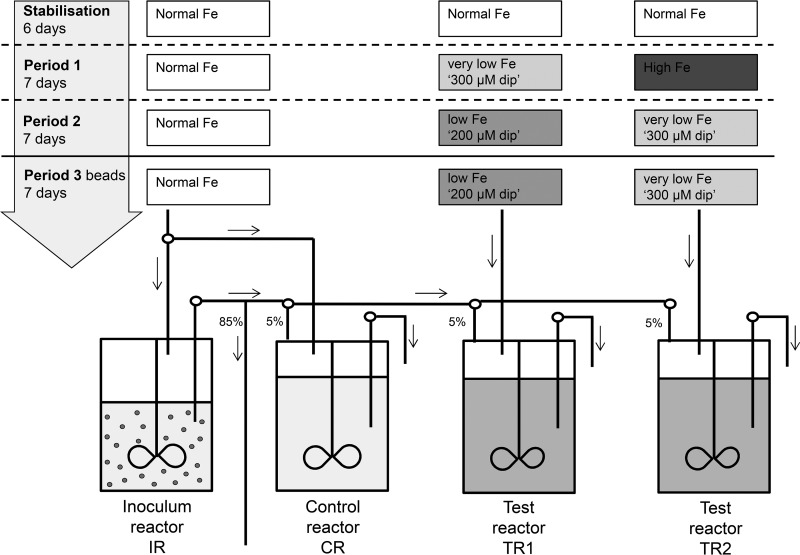
Experimental setup used in this study. Continuous colonic *in vitro* fermentation model PolyFermS, with a first-stage inoculum reactor containing beads with immobilized child gut microbiota and second-stage control and test reactors operated in parallel and continuously inoculated with effluent from the inoculum reactor at 5% (vol/vol) of the reactor volume. Ninety-five percent fresh medium containing different concentrations of Fe was continuously added during periods 1 to 3. In period 3, the inoculum reactor was stopped and the fecal microbiota beads were divided among the control and test reactors.

**FIG 2:**
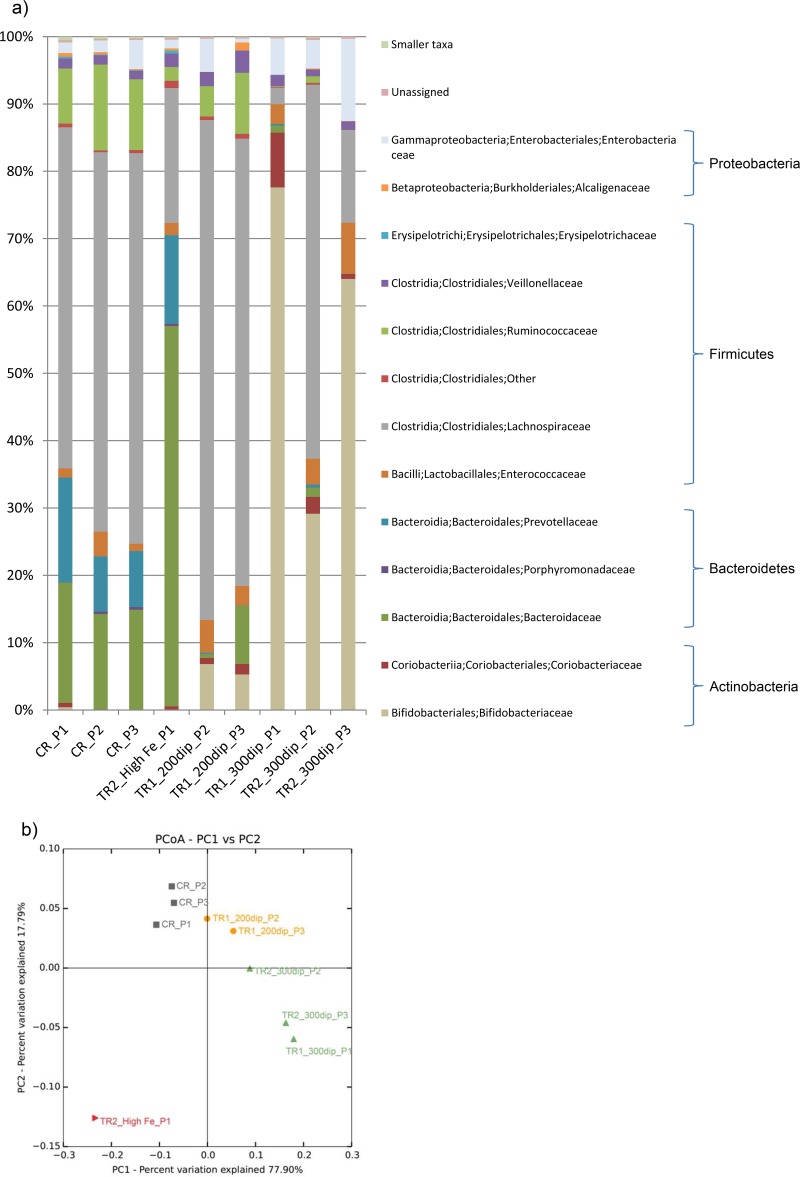
Changes in bacterial communities and diversities in reactor effluents during application of medium differing only in Fe availability. 16S rRNA genes were sequenced using Roche 454 pyrosequencing and analyzed with QIIME and SILVA. (a) Relative abundances of 16S rRNA genes annotated on the family level. (b) Principal coordinate analysis (PCoA) of microbial community β-diversity reveals shifts due to the application of medium differing in Fe availability. P1, P2, and P3, periods 1, 2, and 3; CR, control reactor; TR1 and TR2, treatment reactors 1 and 2. High-Fe (217.8 mg Fe liter^−1^ fermentation medium), low-Fe (200dip; Fe chelated with 200 µM 2,2′-dipyridyl in the fermentation medium), and very-low-Fe (300dip; Fe chelated with 300 µM 2,2′-dipyridyl in the fermentation medium) conditions are indicated.

A PolyFermS continuous colonic fermentation model consisting of one inoculum reactor (IR), one control reactor (CR), and two test reactors (TR1 and TR2) was set up to mimic the conditions in the proximal colon of a child, as described in detail in Materials and Methods and in Fig. 1. The inoculum reactor, containing immobilized gut microbiota, was used to continuously inoculate the control reactor, which received normal-Fe medium (5.0 mg·liter^−1^ FeSO_4_ · 7H_2_O), and the test reactors, which received medium with differing Fe concentrations. The gut microbiota compositions in the CR and TRs under different Fe conditions were determined by 16S rRNA gene sequencing analysis of the combined samples from the last 3 days of each of the 3 fermentation periods (Fig. 2a) and by qPCR analysis of specific bacterial groups (see Table S1 in the supplemental material). After an initial stabilization period of feeding normal-Fe medium to all reactors for 6 days, the gut microbiota compositions were highly similar in the IR, CR, TR1, and TR2 as assessed by qPCR (see Table S1), and the composition was stable over time in the CR, where the Fe concentration of the medium was not changed (Fig. 2a; see also Table S1). Changing the Fe concentration in the feed medium affected the microbial community structure profoundly, as observed by 16S rRNA gene-sequencing analysis and qPCR and further confirmed by β-diversity and principal coordinate analysis (PCoA), whose results are displayed in Fig. 2b.

When very-high-Fe conditions were implemented (TR2, high Fe [1.1 g·liter^−1^ FeSO_4_ ⋅ 7H_2_O, corresponding to 217.8 mg Fe·liter^−1^], period 1), a shift toward lower abundances of *Lachnospiraceae* and *Ruminococcaceae* was observed, while *Bacteroidaceae* strongly increased. The sensitivity of these bacterial families to changing Fe concentrations was further demonstrated when low-Fe conditions were mimicked by feeding medium containing 200 µM 2,2′-dipyridyl (referred to hereinafter as 200 µM dip medium) (TR1, 200 µM dip, period 2, and TR1, 200 µM dip, period 3), where the *Bacteroidaceae* and *Prevotellaceae* abundances decreased while that of *Lachnospiraceae* increased compared to the microbial community in the CR. Interestingly, *Bifidobacteriaceae* and *Coriobacteriaceae* seemed to thrive in environments where Fe was scarce, as shown by their increasing abundance when the low-Fe 200 µM dip medium was applied and the even stronger increase in the very-low-Fe medium containing 300 µM 2,2′-dipyridyl (referred to hereinafter as 300 µM dip medium). Further decreasing Fe availability by using the very-low-Fe 300 µM dip medium in the reactors (TR1, 300 µM dip, period 1; TR2, 300 µM dip, period 2; and TR2, 300 µM dip, period 3) enhanced the effects on the bacterial community observed with the low-Fe 200 µM dip and additionally decreased *Lachnospiraceae* and *Ruminococcaceae*, while *Enterobacteriaceae* and *Enterococcaceae* increased. qPCR revealed a decrease especially in the *Roseburia* spp/*E. rectale* group and members of *Clostridium* cluster IV, such as *Faecalibacterium prausnitzii*, during very-low Fe availability in the reactors (see Table S1 in the supplemental material). However, the application of very-low-Fe 300 µM dip medium in TR2 during fermentation period 2 (TR2, 300 µM dip, period 2) resulted in changes similar to those seen with the application of low-Fe 200 µM dip medium and differing from the results for the other two fermentation periods with very-low-Fe 300 µM dip medium (TR1, period 1, and TR2, period 3) (Fig. 2a and b), probably due to residual bioavailable Fe from the previous high-Fe treatment period, as visually observed in a black biofilm on reactor walls. No distinct differences could be detected between the effects of the two levels of Fe deficiency on planktonic bacteria (periods 1 and 2) and sessile bacteria (period 3).

Phylogenetic β-diversity, determined from operational taxonomic units (OTUs) and calculated using weighted UniFrac analysis, revealed changes in bacterial communities due to differing relative abundances and presence of taxa. Visualization of these changes in PCoA plots clearly showed the repeatability of the shifts within the bacterial ecosystem during the application of low-Fe 200 µM dip and very-low-Fe 300 µM dip medium compared with the bacterial ecosystem in the CR, mainly along the first principal component (Fig. 2b).

Because there is an association between phylogenetic classification and bacterial pathway abundance, functional profiles of the different bacterial ecosystems were predicted using 16S rRNA gene sequence information in PICRUSt (see Fig. S1 in the supplemental material). Pathways involved in glycan, energy, and carbohydrate metabolism were predicted to be slightly more abundant within the microbial ecosystem under high-Fe conditions than under normal-Fe conditions (CR). On the other hand, membrane transport pathways were more abundant when very-low-Fe 300 µM dip medium was applied, while energy metabolism seemed to be attenuated, along with cell motility, compared to the energy metabolism and cell motility under normal-Fe conditions.

### Metabolic activity of the gut microbiota in reactors with different Fe conditions.

Gut microbiota metabolic activity was assessed during the entire fermentation by high-performance liquid chromatography (HPLC) analysis of reactor effluent samples, to monitor the stability of the system and measure changes due to Fe availability (Table 1). After the initial stabilization, the main metabolites were acetate (42.8% ± 0.8%, mean percentage of total SCFA ± standard deviation [SD] from CR, TR1, and TR2), butyrate (33.3% ± 1.3%), and propionate (23.9% ± 0.5%), while no lactate or formate was detected. High-Fe conditions (TR2, High Fe, period 1) led to decreased butyrate production, while propionate was significantly increased compared with the levels in the CR, containing normal-Fe medium (Table 1, period 1, TR2). The addition of low-Fe 200 µM dip medium to the reactors repeatedly increased butyrate formation compared with the concentrations in the CR, while the acetate and propionate concentrations were significantly reduced (Table 1). Under these conditions, butyrate became the dominant metabolite, whereas in the CR, acetate was the main metabolite, followed by butyrate and propionate. Simultaneously, lactate and, especially, formate accumulated in the fermentation effluents when low-Fe 200 µM dip medium was used; these SCFA were not detected in the CR. Fermentation under the very-low-Fe availability of 300 µM dip medium led to significantly decreased propionate and butyrate concentrations compared to those in the CR, while acetate was either significantly increased (period 1) or unchanged (period 3). Therefore, acetate was the dominant metabolite and lactate and formate accumulated in large quantities in the very-low-Fe 300 µM dip medium. Similar to the observations from the microbial composition data, feeding very-low-Fe 300 µM dip medium to TR2 during fermentation period 2 resulted in changes in SCFA production similar to the ones observed in the low-Fe 200 µM dip medium and differing from those in the other two fermentation periods with very-low-Fe 300 µM dip medium (TR1, period 1, and TR2, period 3), probably due to residual bioavailable Fe from the previous high-Fe treatment period, as visually observed in a black biofilm on reactor walls.

**TABLE 1:** Concentrations of metabolites measured by HPLC in fermentation effluent samples from the control reactor under normal-Fe conditions and from the test reactors fed with low-Fe or very-low-Fe medium

Fermentation period, reactor, Fe availability^a^	Mean concn (mM) ± SD of^b^:				
	Acetate	Propionate	Butyrate	Lactate	Formate
Stabilization, normal Fe					
CR	47.2 ± 4.3	26.4 ± 2.3	38.4 ± 0.9	ND	ND
TR1	48.7 ± 4.1	27.1 ± 2.3	35.5 ± 2.0*	ND	ND
TR2	48.8 ± 2.4	27.1 ± 2.2	38.6 ± 1.4	ND	ND
Period 1					
CR	59.2 ± 5.0	23.3 ± 2.2	36.0 ± 2.8	ND	ND
TR1, 300 µM dip	69.5 ± 2.0*	1.8 ± 0.1*	3.2 ± 0.2*	7.3 ± 0.8	15.2 ± 0.7
TR2, high Fe	63.5 ± 4.7	32.6 ± 1.3*	25.9 ± 2.8*	ND	ND
Period 2					
CR	68.5 ± 1.2	16.1 ± 1.3	34.7 ± 1.9	ND	ND
TR1, 200 µM dip	36.7 ± 1.8*	7.2 ± 1.5*	42.8 ± 0.8*	3.2 ± 0.4	18.0 ± 0.4
TR2, 300 µM dip	47.5 ± 5.3*	7.0 ± 2.2*	30.3 ± 3.0*	4.6 ± 0.8	18.3 ± 2.2
Period 3					
CR	67.2 ± 2.9	18.4 ± 0.4	35.6 ± 2.3	ND	ND
TR1, 200 µM dip	31.0 ± 1.1*	9.7 ± 1.3*	48.2 ± 2.3*	1.8 ± 1.6	11.5 ± 0.7
TR2, 300 µM dip	68.7 ± 2.5	3.3 ± 0.2*	12.9 ± 1.5*	5.3 ± 0.5	16.2 ± 1.2

^a^ Normal Fe, 5.0 mg·liter^−1^ FeSO_4_ · 7H_2_O; 300 or 200 µM dip, very-low-Fe or low-Fe condition resulting from 300 or 200 µM 2,2′-dipyridyl in the feed medium of the reactor; high Fe, 1.1 g·liter^−1^ FeSO_4_ · 7H_2_O; CR, control reactor; TR1 and TR2, test reactors 1 and 2.

^b^ Values are the results for the combined samples from the last 3 days of each fermentation period. Values marked by an asterisk are significantly different from the value for the control reactor in the same period within the results for each metabolite (*P* < 0.05). ND, not detected.

Because the chyme medium only differed in Fe availability, the efficacy of substrate carbon conversion to major bacterial metabolites could be estimated from the yields of acetate, propionate, butyrate, lactate, and formate concentrations expressed in C-mole (formate, 1× C; acetate, 2× C; lactate 3× C; propionate, 3× C; and butyrate, 4× C). The total carbon outputs in low-Fe 200 µM dip medium (299.1 mM C in period 3 with 200 µM dip) and very-low-Fe 300 µM dip medium (225.7 mM C in period 3 with 300 µM dip) were decreased compared with that in the CR (331.9 mM C).

### Abundance and expression of the butyryl-CoA:acetate CoA-transferase gene in the gut microbiota under different Fe conditions.

**FIG 3:**
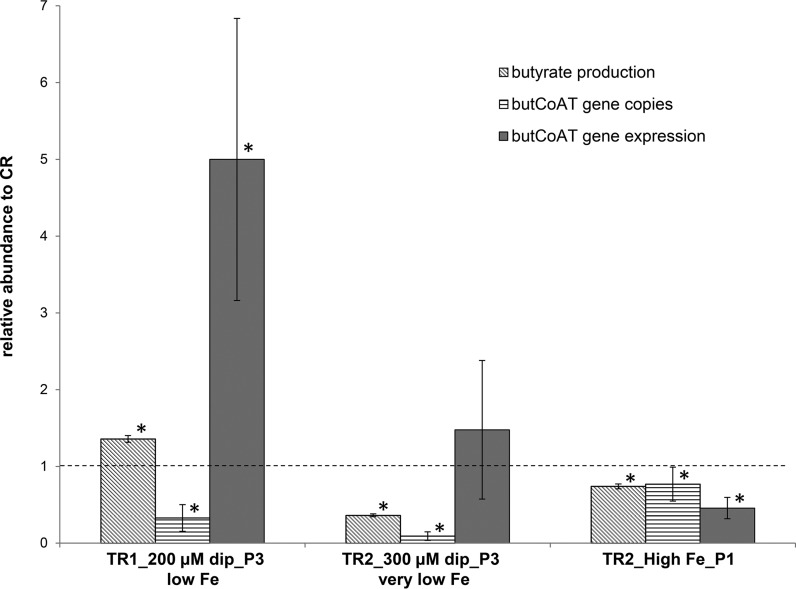
Butyrate concentrations (measured by HPLC), butyryl-CoA:acetate CoA-transferase (*butCoAT*) gene copy numbers (measured by qPCR in total DNA extracts from effluents), and *butCoAT* gene expression levels (measured by qPCR in total RNA extracts from effluents and normalized to 16S rRNA gene expression) in low-Fe 200 µM dip and very-low-Fe 300 µM dip fermentation medium (period 3) and in high-Fe fermentation medium (period 1) were calculated relative to the corresponding data from the control reactor (CR; dotted line). Data are mean results ± SD for the last 3 days of the corresponding fermentation period. Bars marked by an asterisk show values significantly different from the corresponding value for the CR within the same parameter (*P* < 0.05).

Butyryl-CoA:acetate CoA-transferase carries out the last step of butyrate production by using acetate as a cosubstrate while producing butyrate and acetyl-CoA from butyrate-CoA (25–27). In high-Fe medium, significant decreases in the *butCoAT* gene concentration (77% ± 22%) and *butCoAT* gene expression (46% ± 14%) were measured, compared with the gene’s concentration and expression in the CR (100%), together with a reduction in butyrate production (74.0% ± 3.1% of CR) (Fig. 3).

Adding low-Fe 200 µM dip medium to TR1 in period 3 also significantly decreased the *butCoAT* gene concentrations, to 33% ± 17% of the concentration of *butCoAT* genes present in the CR, while in contrast, *butCoAT* gene expression was strongly induced, by 5.0-fold ± 1.8-fold. Hence, the butyrate production was also markedly increased, by 1.4-fold ± 0.04-fold compared to that in the CR.

In very-low-Fe 300 µM dip medium, the abundance of the *butCoAT* gene was decreased to only 9% ± 6% of the abundance found with normal-Fe medium in the CR, while *butCoAT* gene expression (1.5-fold ± 0.9-fold induction) was not significantly different from that in the CR. Under this condition, a decrease in butyrate production to 36.2% ± 1.8% of the butyrate production under normal-Fe conditions in the CR was observed.

### *R. intestinalis* metabolic activity and gene expression under different Fe conditions.

**FIG 4:**
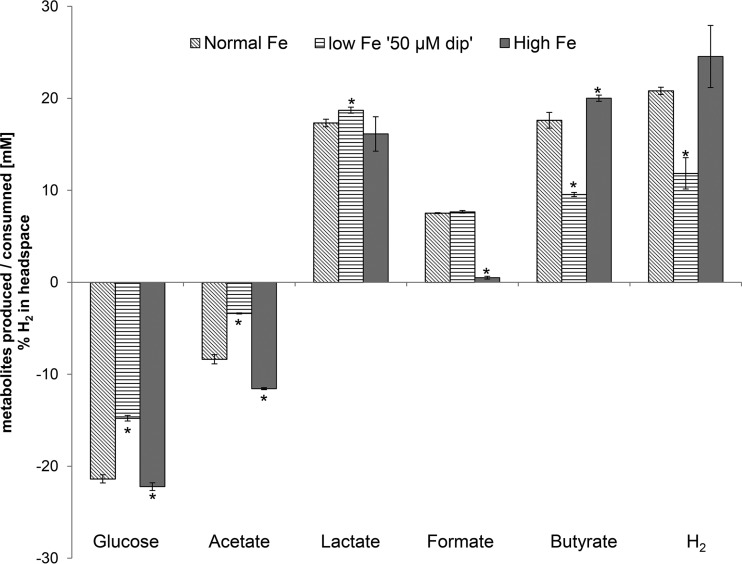
Consumption of glucose and acetate and production of lactate, formate, butyrate, and hydrogen by *R. intestinalis* under normal-Fe, low-Fe (50 µM dip) and high-Fe conditions after 24 h of incubation. Values are mean results ± SD (*n* = 3). Columns marked by an asterisk show values significantly different from the corresponding values for normal-Fe conditions (*P* < 0.05).

*R. intestinalis* DSM14610^T^ was used as a model butyrate producer to investigate the effects of different Fe availabilities on butyrate production using simple single-strain batch cultures in yeast extract-casein hydrolysate-fatty acid (YCFA) medium (Fig. 4; see also Fig. S2 and S3 in the supplemental material). In high-Fe YCFA medium (25 mg Fe liter^−1^ added), the growth behavior was similar to that in normal-Fe YCFA medium (see Fig. S2), but the levels of substrate consumption and metabolite production over time (see Fig. S3) and after 24 h of incubation were different. In high-Fe YCFA medium, significantly more glucose and acetate were consumed and there was a significant increase of butyrate production, while formate production was strongly decreased, compared with normal-Fe YCFA medium. In contrast, the lactate concentration was not changed, resulting in a lactate:butyrate ratio of 1:1.24, compared with a 1:1 ratio in normal-Fe YCFA medium. *R. intestinalis* growth in low-Fe 50 µM dip YCFA medium was similar to that in normal-Fe YCFA medium until 8 h, but the optical density at 650 nm (OD_650_) was significantly lower (1.0 ± 0.0) after 24 h (see Fig. S2). Significantly less glucose and acetate were used in low-Fe 50 µM dip YCFA medium, and the levels of butyrate and H_2_ production were strongly reduced compared with those in normal-Fe YCFA medium (Fig. 4). On the other hand, lactate production was significantly increased, resulting in a 1:0.51 ratio of lactate:butyrate, while the formate excretion was similar to that under normal-Fe conditions. When highly Fe-deficient conditions were mimicked with very-low-Fe 150 µM dip YCFA medium, *R. intestinalis* growth was completely inhibited, and hence, no substrates were used or metabolites produced (see Fig. S2).

**FIG 5:**
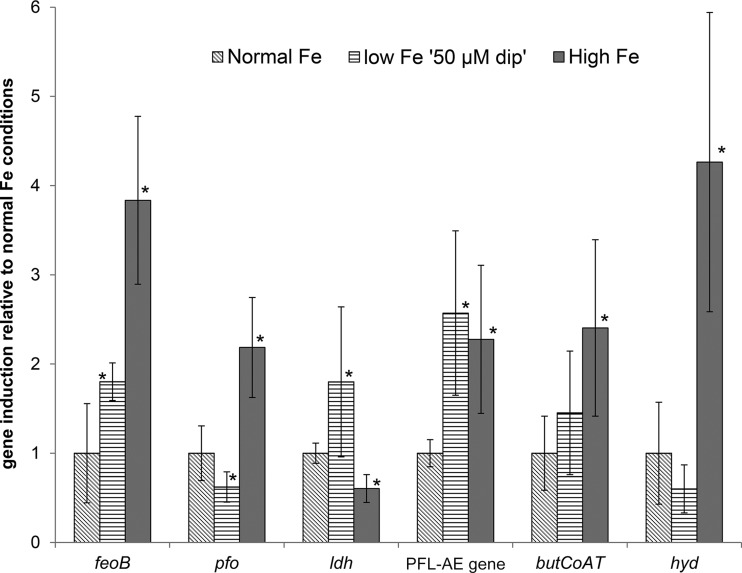
Relative expression levels of the Fe^2+^ transporter gene (*feoB*), pyruvate:ferredoxin-oxidoreductase gene (*pfo*), lactate dehydrogenase gene (*ldh*), pyruvate formate lyase-activating enzyme gene (PFL-AE gene), butyryl-CoA:acetate CoA-transferase gene (*butCoAT*), and hydrogenase gene (*hyd*) in *R. intestinalis* grown under normal-Fe, low-Fe (50 µM dip), and high-Fe conditions. Gene induction was calculated relative to gene expression under normal-Fe conditions. Values are mean results ± SD (*n* = 6). Columns marked by an asterisk show values significantly different from the corresponding values for normal-Fe conditions (*P* < 0.05).

The expression of selected genes encoding enzymes involved in the butyrate production pathway in *R. intestinalis*, such as the pyruvate:ferredoxin-oxidoreductase gene (*pfo*), lactate dehydrogenase gene (*ldh*), pyruvate formate lyase-activating enzyme gene (PFL-AE gene), hydrogenase gene (*hyd*), and *butCoAT* gene, as well as the Fe^2+^ transporter gene (*feoB*), were analyzed by qPCR in cDNA of *R. intestinalis* harvested in exponential growth phase (OD_650_ = 0.6 and 0.7) after incubation in normal-Fe, low-Fe 50 µM dip, and high-Fe (25 mg Fe liter^−1^ added) YCFA medium (Fig. 5). In high-Fe YCFA medium, most genes were induced compared with their expression in normal-Fe YCFA medium, as follows: *feoB*, 3.8-fold ± 0.9-fold; *pfo*, 2.2-fold ± 0.6-fold; the PFL-AE gene, 2.3-fold ± 0.8-fold; *butCoAT*, 2.4-fold ± 1.0-fold; and *hyd*, 4.2-fold ± 1.7-fold. In contrast, *ldh* was significantly downregulated (0.6-fold ± 0.2-fold) in high-Fe YCFA medium. In low-Fe 50 µM dip YCFA medium, *feoB* (1.8-fold ± 0.2-fold), the PFL-AE gene (2.3-fold ± 0.9-fold), and *ldh* (1.8-fold ± 0.8-fold) expression was significantly induced, while *pfo* expression was decreased (0.6-fold ± 0.2-fold).

## DISCUSSION

Our *in vitro* fermentation studies using immobilized child gut microbiota and the model butyrate producer *R. intestinalis* were designed to provide a mechanistic insight into the influence of Fe availability on the butyrate production capacity. Our data confirmed the results from our previous *in vitro* fermentation study (15) showing that Fe availability strongly modulated the metabolic activity of the gut microbiota and *R. intestinalis*. Interestingly, we found that under low-Fe conditions (200 µM 2,2′-dipyridyl), butyrate producers in a complex ecosystem had a growth advantage, while under very-low-Fe conditions (300 µM 2,2′-dipyridyl), butyrate production was impaired. Moreover, very-high Fe concentrations caused a shift in the gut microbial ecosystem, as well as in metabolites, while on the single-strain level, the butyrate production of *R. intestinalis* was increased.

After the initial stabilization period of the PolyFermS colonic *in vitro* continuous fermentation model, all reactors exhibited a highly similar gut microbiota composition and metabolic activity, representing a stable and diversified microbiota, in agreement with previous studies (15, 22–24).

Under high-Fe fermentation conditions, the increased abundance of propionate-producing *Bacteroidaceae* (29) and decreased abundance of butyrate-producing *Lachnospiraceae* (25) were also reflected in the metabolic activity, with an increase in propionate production and a decrease in butyrate production, along with a lower number of *butCoAT* genes present and expressed. In contrast, a previous fermentation experiment with a lower concentration of supplemented Fe (26.5 mg Fe liter^−1^) showed no major effect on the gut microbiota composition and metabolic activity (15). In experiments with bioreactors for H_2_ production using strict anaerobic cultures, butyrate formation was also impaired with very-high Fe concentrations (30–32). These data suggest that there is an optimal Fe concentration for butyrate production by the microbial ecosystem, beyond which a toxic effect of Fe may occur (2).

Data obtained under conditions of Fe deficiency in this study (low-Fe 200 µM dip medium and very-low-Fe 300 µM dip medium) have a high degree of correlation with the results from our previous colonic *in vitro* fermentations mimicking Fe deficiency and those of a rat study with severely Fe-deficient rats (14, 15). Mimicking Fe deficiency in the reactors repeatedly caused shifts in the microbial community and affected the dominant bacterial groups*.* While *Bacteroidaceae* were strongly reduced and *Bifidobacteriaceae* promoted by both levels of Fe deficiency, *Enterobacteriaceae* remained unaffected. *Enterobacteriaceae* and bifidobacteria are reported to be very good Fe scavengers and, thus, likely have a growth advantage in Fe-limited environments (1, 33). *Lachnospiraceae*, the *Roseburia* spp./*E. rectale* group, and *Eubacterium hallii* seemed unaffected by low-Fe conditions (200 µM dip) but were reduced under very-low-Fe conditions (300 µM dip).

These observed changes in microbial composition during Fe deficiency were also reflected by differences in gut microbiota metabolic activity. Propionate was decreased during Fe deficiency, likely explained by a decrease in the propionate-producing bacterial clade of *Bacteroidaceae* (29). Butyrate production was strongly impaired under very-low-Fe conditions (300 µM dip), correlating with a decrease in butyrate-producing members of *Lachnospiraceae*. However, the increase of butyrate production during low-Fe conditions (200 µM dip), along with a decrease of acetate production by 50%, was unexpected. Therefore, we investigated the gene expression of *butCoAT*, which mediates the last step in the butyrate production pathway by transferring the CoA unit from butyryl-CoA to acetate and releasing butyrate (25, 34). Although the *butCoAT* copy numbers per milliliter of effluent were decreased in low-Fe 200 µM dip medium, we found that the *butCoAT* gene was strongly overexpressed. Moreover, up to 85% of butyrate production can be derived from acetate utilization by butyrate producers possessing the product of *butCoAT* as a final enzyme in the butyrate production pathway (34). This may explain the increase in butyrate and the decrease in acetate in low-Fe 200 µM dip medium compared with their concentrations under normal-Fe conditions. Moreover, our data suggest that Fe not only influences metabolic activity as a cofactor in Fe-dependent enzymes involved in the butyrate production pathway, such as hydrogenases and oxidoreductases (25, 35), but also regulates the *butCoAT* gene expression of butyrate producers. The production of lactate and formate, which can be side products during butyrate formation (25), was increased under low-Fe conditions, suggesting that other genes in the butyrate production pathway may also be up- or downregulated by differing Fe levels.

Although the combined total carbohydrate conversion was decreased under highly Fe-deficient conditions, the predicted prevalence of functional pathways involved in carbohydrate and energy metabolism were not markedly decreased within the microbiome. This shows that although pathways for carbohydrate conversion are present, Fe is needed for an efficient conversion of dietary complex carbohydrates into absorbable metabolites, which leads to further energy extraction from the diet (19).

**FIG 6:**
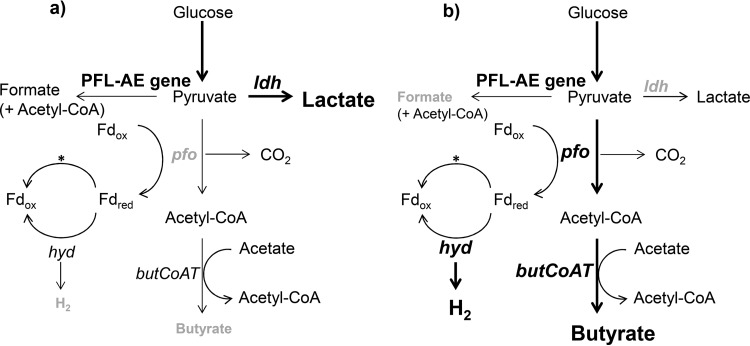
Schematic view of genes and metabolites in the butyrate production pathway of *R. intestinalis* grown in low-Fe 50 µM dip (a) and in high-Fe (b) YCFA medium. The pyruvate:ferredoxin-oxidoreductase gene (*pfo*), lactate dehydrogenase gene (*ldh*), pyruvate formate lyase-activating enzyme gene (PFL-AE gene), butyryl-CoA:acetate CoA-transferase gene (*butCoAT*), and hydrogenase gene (*hyd*) are depicted in gray to show reduced expression or in boldface to show increased induction compared with their expression in normal-Fe YCFA medium. Metabolites are depicted as decreased (gray) or increased (boldface) compared with their levels in normal-Fe YCFA medium. *, the regeneration of reduced ferredoxin to the oxidized form is also processed by a membrane-associated NADH:ferredoxin-oxidoreductase (adapted from references 25, 35, and 37).

The modulations of the gut microbiota due to Fe availability, and especially the changes in butyrate production, reported in this and other studies (14–16), led us to the hypothesis that butyrate producers are highly responsive to differing Fe levels in the environment. Therefore, we investigated the effects of differing Fe levels on the metabolic activity and expression of selected genes in the butyrate production pathway of *R. intestinalis* DSM14610^T^, an important butyrate producer in the gut (25, 36). The observed changes are summarized in Fig. 6a and b. Our results suggest that pyruvate:ferredoxin oxidoreductase (*pfo*), which converts pyruvate to acetyl-CoA by reducing ferredoxin (37, 38), might play a key role in the *R. intestinalis* response to different Fe levels. This enzyme also depends on a membrane-associated hydrogenase (*hyd*) that regenerates reduced ferredoxin to oxidized ferredoxin (39). In low-Fe 50 µM dip YCFA medium, *pfo* expression was downregulated, and hence, the conversion from pyruvate to acetyl-CoA was limited (Fig. 6a). This effect could have been enhanced by the impaired regeneration of ferredoxins by the hydrogenase, which depends on Fe as a cofactor (40). Indeed, in low-Fe 50 µM dip YCFA medium, H_2_ production by *R. intestinalis* was strongly impaired, although *hyd* expression was not reduced. Similar observations were made for the strictly anaerobic butyrate producer *Clostridium acetobutylicum*, whose *hyd* gene expression was unchanged under Fe-limited conditions (4). Moreover, the expression of the *ldh* and PFL-AE gene was upregulated in low-Fe 50 µM dip YCFA medium, leading to enhanced conversion of pyruvate from glycolysis to formate and lactate under Fe-limited conditions, as observed in the *in vitro* colonic fermentation experiment. Lactate was also reported as the predominant metabolic product in *C. acetobutylicum* under Fe-limited conditions (41), and an increase in *ldh* expression of *C. acetobutylicum* under Fe-restricted conditions was shown (4). The observed changes in metabolic conversion of pyruvate were associated with a lower biomass production of *R. intestinalis* under low-Fe conditions. This has already been observed for *Clostridium difficile*, also a strictly anaerobic hydrogen producer, under low-Fe conditions generated by the addition of 2,2′-dipyridyl to the growth medium (42).

In high-Fe YCFA medium, however, *pfo* and, also, *hyd* expression, as well as *butCoAT* expression, were induced, along with a reduced expression of *ldh*, which resulted in pyruvate being preferentially transformed into acetyl-CoA and, ultimately, into butyrate (Fig. 6b). This was also visible by an increase in H_2_ formation. In bioreactors for hydrogen production by mixed cultures of strict anaerobes, the addition of moderate amounts of Fe also led to increased butyrate and hydrogen production (30–32). However, the same study also showed that further increases in Fe concentrations led to decreased butyrate and H_2_ production, in agreement with our *in vitro* gut fermentation data.

The data for *R. intestinalis* grown in low-Fe 50 µM dip YCFA medium support the findings of this and previous colonic *in vitro* fermentation experiments (15). The final metabolites under very-low-Fe conditions were shaped toward lower butyrate concentrations, while lactate and formate were produced preferentially, probably due to a restricted functionality of the combined action of a pyruvate:ferredoxin oxidoreductase and hydrogenase in some butyrate producers. This metabolic shift may have negative impacts on gut health due to the lack of beneficial butyrate and the accumulation of lactate. Butyrate has many health-promoting effects, such as anticancer and anti-inflammatory properties, and is an energy source for enterocytes (20, 21). Furthermore, the accumulation of lactate has been associated with inflammatory bowel disease and ulcerative colitis (43, 44).

The present study highlights the crucial role of Fe availability not only in the complex bacterial ecosystem of the human gut but also at the single-strain level, especially for butyrate production. Our study shows that the gut microbiota requires sufficient Fe to optimally maintain its metabolic function, even though predicted functional pathways may be present in the bacterial ecosystem. End fermentation products of the gut microbiota, such as propionate and butyrate, were decreased under low-Fe conditions, leading to a reduced carbon conversion efficiency, which may result in impaired extraction of energy from the diet. Butyrate production seems to be strongly connected to Fe availability, and there may be an optimal Fe level for health-promoting butyrate production in the colon. More investigations are needed, especially *in vivo* studies, to understand the complex interplay between the gut microbiota, Fe, and the host.

## MATERIALS AND METHODS

### Continuous colonic fermentation setup.

The PolyFermS continuous colonic fermentation model used in this study was adapted from the *in vitro* colonic fermentation models described previously (22, 23) and was designed to mimic the conditions in the proximal colon of a child (Fig. 1). Briefly, the fermentation setup consisted of an inoculum reactor (IR), which contained immobilized child gut microbiota, and three subsequent reactors, which were continuously inoculated with fermentation effluent from the inoculum reactor, aiming to generate the same microbiota in control and test reactors. The inoculum reactor and control reactor (CR) received normal-Fe medium during the entire fermentation, while medium with differing Fe concentrations was used in the test reactors (TR1 and TR2). The fermentation was divided into a stabilization period, where a stable microbiota was allowed to establish in the IR, CR, and TRs, and 3 fermentation periods, where medium differing only in the Fe concentrations was added to the reactors (Fig. 1). During fermentation period 3, the beads in the IR containing immobilized gut microbiota were harvested and divided equally among the CR, TR1, and TR2 in order to assess the effects of different Fe availabilities on sessile gut microbiota.

### Gut microbiota immobilization, fermentation procedures, medium design, and sampling.

A fresh fecal sample from a healthy 2.5-year-old child, with a diversified gut microbiota and not receiving antibiotics for the previous 3 months, was collected anaerobically and immobilized in gellan-xanthan beads within 2 h of defecation, as described previously (15, 45, 46). The Ethics Committee of ETH Zurich exempted this study from review because fecal sample collection was not in terms of intervention. An informed written consent was, however, obtained from the fecal donors. Fermentation mimicking the proximal colon of a child was carried out by constantly flushing the headspace of all reactors (Sixfors; Infors, Switzerland) with CO_2_ to maintain anaerobiosis, setting the pH at 5.7 by the addition of 2.5 M NaOH, and keeping the temperature at 37°C. Beads inoculated with fecal microbiota were colonized in the inoculum reactor for 48 h, while the medium was exchanged every 12 h. The inoculum reactor (total fermentation volume of 200 ml) was switched to continuous mode at a flow rate of 25 ml h^−1^, resulting in a medium inflow/outflow of 600 ml in 24 h and a retention time of 8 h (47). After 4 days, the CR, TR1, and TR2 (volume of 210 ml each reactor) were connected and operated in continuous mode at an inflow rate of 25 ml h^−1^ fresh medium and 1.25 ml h^−1^ effluent from the IR, which contained gut microbiota for inoculation, for a total retention time of 8 h.

The fermentation medium used was similar to a previously described medium that mimics the chyme reaching the proximal colon of a child (15, 45, 48, 49) and is described in detail in the supplemental material. The medium was modified using the ferrous Fe chelator 2,2′-dipyridyl (Sigma-Aldrich, Switzerland) to mimic Fe deficiency (200 µM 2,2′-dipyridyl [low-Fe 200 µM dip medium]) and strong Fe deficiency (300 µM 2,2′-dipyridyl [very-low-Fe 300 µM dip medium]7). 2,2′-Dipyridyl is a highly specific Fe chelator that is widely used to generate Fe-deficient conditions in bacterial growth media (42, 50). Strong Fe supplementation was mimicked by the addition of 1.1 g·liter^−1^ FeSO_4_ ⋅ 7H_2_O (217.8 mg Fe liter^−1^) to the fermentation medium (high-Fe medium), for a total daily Fe supplementation of 130.7 mg Fe (600-ml medium inflow within 24 h). This corresponds to approximately twice the maximum recommended Fe supplementation in children of 60 mg per day (51, 52), which we chose for the mechanistic objective of the study. FeSO_4_ is highly bioavailable and the gold standard in Fe supplementation (5).

Sampling was performed daily, and samples for qPCR and RNA and pyrosequencing analysis were frozen at −80°C. Effluent samples for SCFA analysis were centrifuged (10,000 × *g* for 10 min), and the supernatant was used immediately for HPLC analysis.

### Genomic DNA extraction and qPCR procedures.

The FastDNA spin kit for soil (MP Biomedicals), with a bead-beating step to lyse cells, was used to extract genomic DNA from 1.5 ml of fermentation effluent from the last 3 days of each fermentation period. An ABI Prism 7500 PCR sequence detection system (Life Technologies, Switzerland) was used to enumerate specific bacterial groups and species prevalent in the gut, using the primers listed in Table S2 and the methods detailed in the supplemental material. Each sample was analyzed in duplicate.

### 16S rRNA gene sequencing analysis and predictive functional profiling.

Effluent samples from the last 3 days of the treatment periods (periods 1 to 3) were combined for each reactor (CR, TR1, and TR2), and genomic DNA was extracted using the FastDNA spin kit for soil (MP Biomedicals, France). Sequencing of the V5-V6 region of the 16S rRNA gene was performed by DNAVision (Charleroi, Belgium) using a 454 Life Sciences genome sequencer FLX (Roche, Switzerland) as described previously (16). For taxonomic sequence analysis, the open source software QIIME (version 1.8.0) (53) and the rRNA database project SILVA (http://www.arb-silva.de) (54) were used according to the standard instructions on QIIME. Sequences were quality trimmed for scores below 25, resulting in 9,654 ± 950 (mean ± SD) reads per sample, before being clustered into OTUs at the 97% identity level with UCLUST (55). In QIIME, representative OTUs were then picked, using open-reference OTU picking, and filtered, and taxonomic assignment was performed against the SILVA database (version 111). For phylogenetic β-diversity analysis in QIIME, which reveals differences between bacterial communities due to differing relative abundances and presence of taxa, each OTU data set was subsampled to 1,000 16S rRNA gene reads per sample. The distance metrics obtained were then visualized using PCoA in QIIME with weighted UniFrac.

The prediction of the abundance of functional pathways in a microbial community with the available 16S rRNA gene sequence information was performed using PICRUSt (phylogenetic investigation of communities by reconstruction of unobserved states) version 1.0.0 (http://picrust.github.io/picrust/) (56). The OTUs were closed reference picked against the Greengenes database (version 13.5) (57) in QIIME and normalized as described previously (58). The resulting data were then imputed in PICRUSt, allowing the prediction of the functional pathways present based on the Kyoto Encyclopedia of Genes and Genomes (KEGG).

### Metabolite analysis.

The concentrations of acetate, butyrate, propionate, formate, and lactate in fermentation effluents were determined by HPLC. The supernatants of fermentation effluents were diluted 1:1 with MilliQ water and subjected to HPLC analysis as described previously (59). Each sample was analyzed in duplicate.

### RNA extraction and gene expression analysis in fermentation effluents.

Effluent samples from the last 3 days of representative fermentation periods for the low-Fe-availability fermentation conditions of 200 µM dip (period 3, TR1) and 300 µM dip (period 3, TR2) and for the high-Fe fermentation condition (period 1, TR2), as well as the corresponding effluents from the CR, were analyzed for *butCoAT* gene expression. This resulted in a triplicate analysis of each condition. The RNA extraction procedures and cDNA transcription are detailed in Text S1 in the supplemental material. cDNA was subjected to qPCR analysis as described above, and *butCoAT* gene expression and total 16S rRNA gene (*rrs*) expression were assessed quantitatively; *butCoAT* expression was normalized to *rrs* expression (absolute copy numbers) and *butCoAT* induction was calculated relative to the *butCoAT* expression in the CR.

### *R. intestinalis* growth and metabolic activity under different Fe conditions.

*Roseburia intestinalis* strain L1-82 (DSM14610^T^) was used as a butyrate-producing model organism (25, 36) and was routinely maintained in YCFA medium (60) (see Text S1 in the supplemental material). The YCFA medium was adapted to generate low-, very-low-, and high-Fe conditions by adding 50 µM 2,2′-dipyridyl (50 µM dip) or 150 µM 2,2′-dipyridyl (50 µM dip) to deplete YCFA medium of bioavailable Fe or by adding 125 mg liter^−1^ FeSO4 ⋅ 7H_2_O (25 mg Fe liter^−1^) to generate high-Fe YCFA medium, respectively. YCFA medium was prepared with 6 g liter^−1^ glucose and without hemin for all Fe conditions. Three separate overnight culture tubes of *R. intestinalis* cultures were used to inoculate each tested medium in triplicate. Growth, metabolites, and gas production were assessed in separate tubes for each time point at 0, 4, 6, 8, and 24 h of incubation at 37°C. Growth was assessed by OD_650_ measurement, and H_2_ accumulation in the headspace of Hungate tubes was measured by gas chromatography (model 5890 series II; Hewlett-Packard, USA). The substrate and metabolite concentrations in culture supernatants were measured by HPLC as described above.

### RNA extraction and gene expression analysis.

*R. intestinalis* cultures (10 ml in Hungate tubes) were grown anaerobically in normal-Fe YCFA medium, 50 µM dip YCFA medium, and high-Fe YCFA medium to late exponential phase at OD_650_ values of 0.6 and 0.7, in triplicate for each OD. The cultures were centrifuged (5,000 × *g* for 5 min) and transferred to an anaerobic chamber, and the pellets were dissolved in 2 ml RNAProtect for bacteria (Qiagen, Switzerland), centrifuged, and then shock-frozen in liquid nitrogen and stored at −80°C until RNA extraction. RNA extraction and cDNA preparation were conducted as described above. Primers were designed for the Fe^2+^ transporter gene (*feoB*) and genes in the butyrate production pathway of *R. intestinalis*, such as the pyruvate:ferredoxin-oxidoreductase gene (*pfo*), lactate dehydrogenase gene (*ldh*), pyruvate formate lyase-activating enzyme gene (PFL-AE gene), and hydrogenase gene (*hyd*). Primer design and the qPCR procedures are described in Text S1 in the supplemental material.

The expression of each gene was normalized to *rrs* gene expression, and gene induction was calculated relative to the gene expression in normal-Fe YCFA medium.

### Statistical analysis.

Statistical analyses were done using JMP 10.0 (SAS Institute, United States). All data except the 16S rRNA gene sequencing data are expressed as the mean results ± SD for the last 3 days of each fermentation period or for triplicate analyses of *R. intestinalis* growth and gene expression. For the *in vitro* gut fermentation experiment, metabolites (HPLC), bacterial composition (qPCR), and gene expression were compared pairwise between the CR and TRs within each fermentation period, using the nonparametric Kruskal-Wallis test. Bacterial composition data (qPCR) were log_10_ transformed before statistical analysis. For HPLC data, growth, and gene expression in the *R. intestinalis* experiments, pairwise comparisons between the results for normal-Fe YCFA medium and 50 µM dip YCFA medium and the results for 150 µM dip YCFA medium and high-Fe YCFA medium were performed using the nonparametric Kruskal-Wallis test. *P* values of <0.05 were considered significant.

### Nucleotide sequence accession number.

The sequence data obtained by sequencing of the V5-V6 region of the 16S rRNA gene have been submitted to the Sequence Read Archive (SRA) of NCBI (https://www.ncbi.nlm.nih.gov/sra) under accession number PRJNA265104.

## SUPPLEMENTAL MATERIAL

Figure S1  Predicted relative abundances of gene functions within the microbial communities under differing Fe availability. Predictions were based on OTUs associated with Greengenes identifiers and imputed using PICRUSt to determine KEGG pathway abundances. Pathways specific for human metabolism (organismal systems and human disease), which were all below 0.5% abundance, were not included in the graph. Download Figure S1, PDF file, 0.03 MB

Figure S2  Growth curve of *R. intestinalis* under normal-Fe and low-Fe conditions generated by the addition of either 50 or 150 µM 2,2′-dipyridyl and under the high-Fe condition (25 mg Fe·liter^−1^). Values are means ± SD (*n* = 3). Values marked by an asterisk are significantly different from the corresponding values for growth under normal-Fe conditions at the same time point (*P* < 0.05). Download Figure S2, PDF file, 0.02 MB

Figure S3  Consumption of glucose and acetate and production of lactate, formate, butyrate, and hydrogen by *R. intestinalis* in normal-Fe (a), low-Fe (50 µM dip) (b), and high-Fe (c) YCFA medium over time. Values are mean results ± SD (*n* = 3). Download Figure S3, PDF file, 0.02 MB

Table S1  16S rRNA gene concentrations (log_10_ copy number ml^−1^ fermentation effluent) of specific bacterial groups and species, determined by qPCR in samples of effluent during normal-Fe conditions in the control reactor (CR) and different Fe conditions in test reactors (TR1 and TR2). Table S1, PDF file, 0.02 MB

Table S2  Primers used to enumerate specific bacterial groups and quantify gene expression by qPCR. Table S2, PDF file, 0.02 MB

Text S1  Supplemental materials and methods. Download Text S1, PDF file, 0.04 MB
