# Supplementary material for: Iron Modulates Butyrate Production by a Child Gut Microbiota In Vitro
Source: mBio. 2015 Nov 17;6(6):e01453-15. doi: 10.1128/mBio.01453-15 (PMC4659462; doi:10.1128/mBio.01453-15)
Supplement: Figure S1 — Predicted relative abundances of gene functions within the microbial communities under differing Fe availability. Predictions were based on OTUs associated with Greengenes identifiers and imputed using PICRUSt to determine KEGG pathway abundances. Pathways specific for human metabolism (organismal systems and human disease), which were all below 0.5% abundance, were not included in the graph. Download [file mbo005152539sf1.pdf]

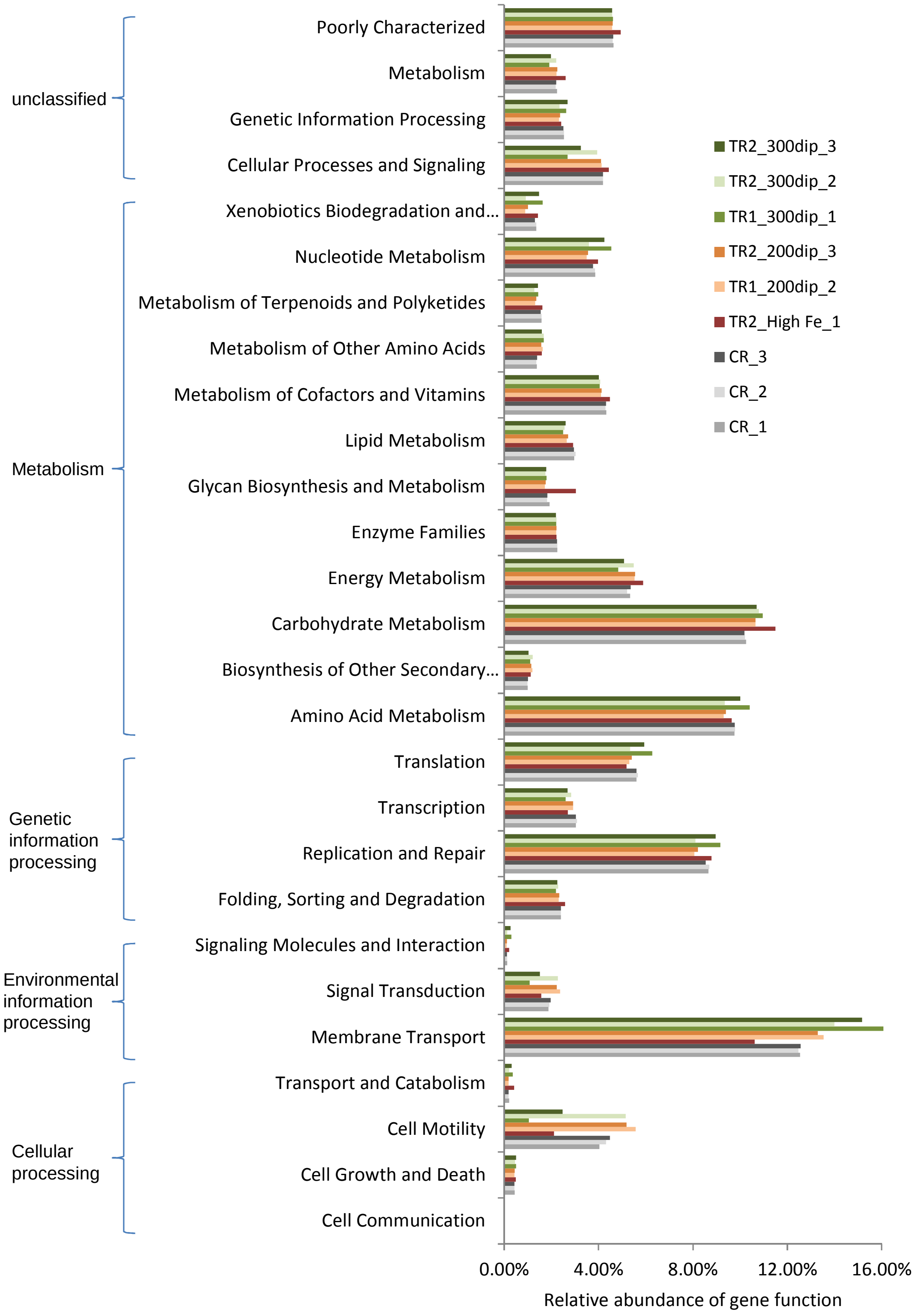

Supplementary Figure S1: Predicted relative abundance of gene functions within the microbial communities under ‘Normal Fe’(CR), ‘High Fe’, low Fe ‘200 μM dip’ and very low Fe ‘300 μM dip’ (TR1 and TR2) conditions. Predictions were based on OTUs associated with Greengenes identifiers and imputed using PICRUSt to determine KEGG pathway abundances. Pathways specific for human metabolism (organismal systems and human disease), which were all below 0.5 % abundance, were not included in the graph.
