# Supplementary material for: Iron Modulates Butyrate Production by a Child Gut Microbiota In Vitro
Source: mBio. 2015 Nov 17;6(6):e01453-15. doi: 10.1128/mBio.01453-15 (PMC4659462; doi:10.1128/mBio.01453-15)
Supplement: Figure S2 — Growth curve of R. intestinalis under normal-Fe and low-Fe conditions generated by the addition of either 50 or 150 µM 2,2′-dipyridyl and under the high-Fe condition (25 mg Fe·liter−1). Values are means ± SD (n = 3). Values marked by an asterisk are significantly different from the corresponding values for growth under normal-Fe conditions at the same time point (P < 0.05). Download [file mbo005152539sf2.pdf]

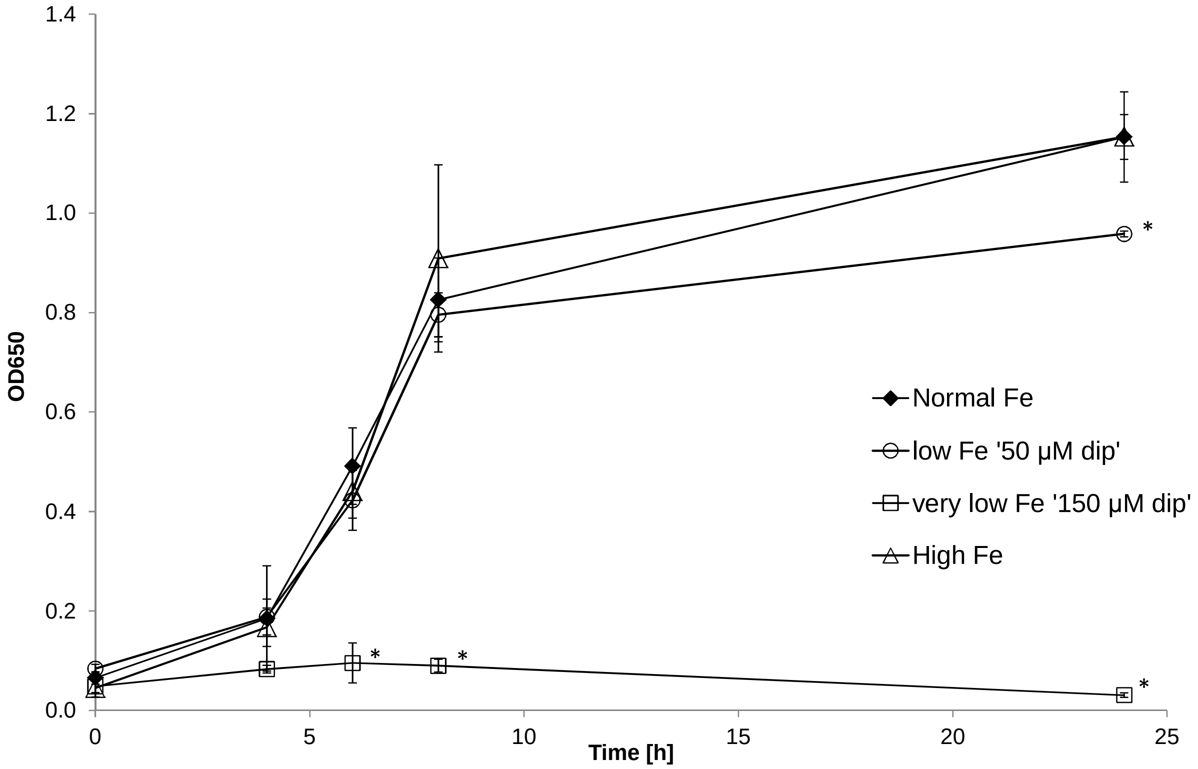

Supplementary Figure S2: Growth curve of *R. intestinalis* in ‘Normal Fe’ YCFA, low Fe ’50 µM dip’ YCFA or very low Fe ‘150 µM dip’ YCFA (addition of 2,2’-dipyridyl) and ‘High Fe’ YCFA (25 mg Fe L^{-1}). Values are means ± SD (n=3). Values with an asterisk (*) are significantly different from growth under normal Fe conditions at the same timepoint, *P* < 0.05.
