# Supplementary material for: Iron Modulates Butyrate Production by a Child Gut Microbiota In Vitro
Source: mBio. 2015 Nov 17;6(6):e01453-15. doi: 10.1128/mBio.01453-15 (PMC4659462; doi:10.1128/mBio.01453-15)
Supplement: Figure S3 — Consumption of glucose and acetate and production of lactate, formate, butyrate, and hydrogen by R. intestinalis in normal-Fe (a), low-Fe (50 µM dip) (b), and high-Fe (c) YCFA medium over time. Values are mean results ± SD (n = 3). Download [file mbo005152539sf3.pdf]

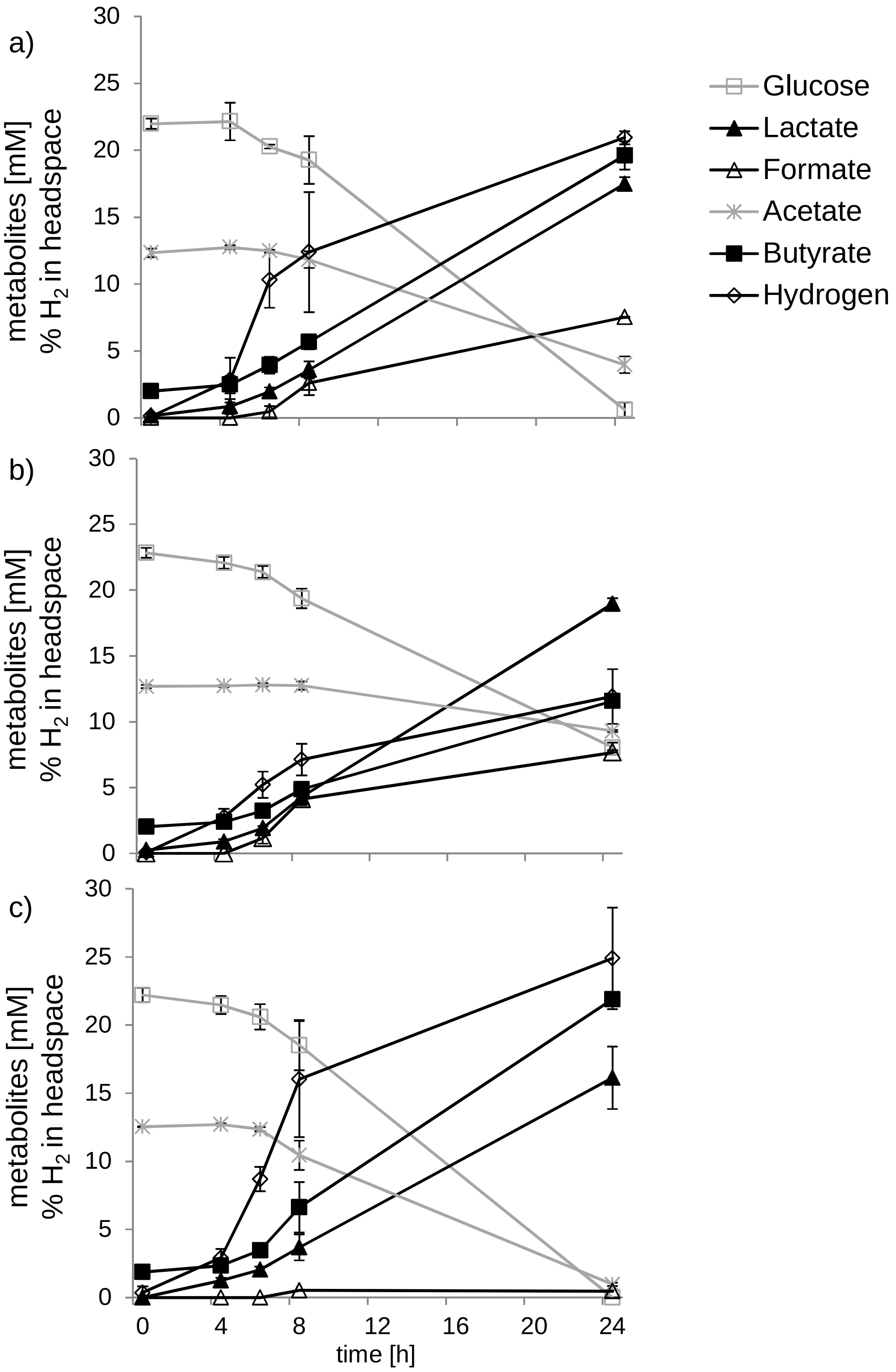

Supplementary Figure S3: Consumption of glucose and acetate and production of lactate, formate, butyrate and hydrogen of *R. intestinalis* in 'Normal Fe' (a), low Fe '50 µM dip' (b) and 'High Fe' (c) YCFA medium over time. Values are means ± SD (n=3).
