# Supplementary material for: Iron Modulates Butyrate Production by a Child Gut Microbiota In Vitro
Source: mBio. 2015 Nov 17;6(6):e01453-15. doi: 10.1128/mBio.01453-15 (PMC4659462; doi:10.1128/mBio.01453-15)
Supplement: Table S1 — 16S rRNA gene concentrations (log10 copy number ml−1 fermentation effluent) of specific bacterial groups and species, determined by qPCR in samples of effluent during normal-Fe conditions in the control reactor (CR) and different Fe conditions in test reactors (TR1 and TR2). [file mbo005152539st1.pdf]

Supplementary Table S1: 16S rRNA gene concentrations (log_{10} copy numbers mL^{-1} fermentation effluent) of specific bacterial groups and species determined by qPCR in effluent samples during 'Normal Fe' conditions in the control reactor (CR) and 'High Fe', low Fe '200 µM dip' and very low Fe '300 µM dip' conditions in test reactors (TR1 and TR2).

	**total 16S**	***Bacteroides* spp.**	**Firmicutes**	***Roseburial E. rectale***	***Clostridium* Cluster IV**	***F. prausnitzii***	***E. hallii***	***Bifidobacterium* spp.**	***Enterobacteriaceae***	**lactobacilli**
**Stabilization**										
CR	11.0 ± 0.04	10.7 ± 0.1	10.6 ± 0.02	10.1 ± 0.1	8. 1 ± 0.1	n.d.	6.4 ± 0.2	7.7 ± 0.3	8.1 ± 0.2	6.0 ± 0. 1
TR1	10.6 ± 0.6	10.5 ± 0.7	10.2 ± 0.6	10.1 ± 0.1	7.7 ± 0.7	n.d.	6.3 ± 0.1	7.5 ± 0.4	7.7 ± 0.4	6.1 ± 0.1
TR2	10.9 ± 0.03	10.7 ± 0.1	10.6 ± 0.1	10.1 ± 0.1	8.0 ± 0.1	n.d.	6.5 ± 0.2	7.7 ± 0.3	7.3 ± 0.1	6.2 ± 0. 1
**Period 1**										
CR	11.0 ± 0.1	10.6 ± 0.1	10.7 ± 0.1	10.2 ± 0.1	10.1 ± 0.2	6.3 ± 0.2	7.6 ± 0.3	7.1 ± 0.1	9.4 ± 0.5	6.4 ± 0.1
TR1 - 300 µM[a]	10.7 ± 0.1*	9.1 ± 0.2*	10.2 ± 0.1*	8.9 ± 0.1*	8.0 ± 0.2*	4.1 ± 0.4[b]	6.5 ± 0.1*	10.1 ± 0.04*	9.7 ± 0.8	5.2 ± 0.3*
TR2 - High Fe	10.9 ± 0.1	10.7 ± 0.2	10.4 ± 0.1*	9.8 ± 0.1*	9.5 ± 0.4	5.8 ± 0.5	7.8 ± 0.1	7.0 ± 0.2	8.8 ± 0.4	6.3 ± 0.2
**Period 2**										
CR	11.1 ± 0.02	10.6 ± 0.1	10.8 ± 0.1	10.3 ± 0.1	10.0 ± 0.04	6.4 ± 0.2	7.8 ± 0.1	7.0 ± 0.2	9.9 ± 0.7	6. 3 ± 0.4
TR1 - 200 µM[a]	11.1 ± 0.1	9.5 ± 0.1*	10.9 ± 0.7	10.4 ± 0.1	9.6 ± 0.3*	5.8 ± 0.2*	8.8 ± 0.1*	9.3 ± 0.1*	9.9 ± 0.4	6.1 ± 0.2
TR2 - 300 µM[a]	10.9 ± 0.1*	9.7 ± 0.2*	10.6 ± 0.1*	10.2 ± 0.2	8.5 ± 0.5*	6.6 ± 0.1	8.2 ± 0.1*	9.8 ± 0.1*	9.8 ± 0.7	5. 8 ± 0.2*
**Period 3 - beads**										
CR	10.8 ± 0.2	10.6 ± 0.1	10.8 ± 0.2	10.2 ± 0.1	9.2 ± 0.1	7.0 ± 0.1	7.0 ± 0.2	6.5 ± 0.2	9.9 ± 0.3	5.9 ± 0.1
TR1 - 200 µM[a]	10.9 ± 0.1	10.1 ± 0.2*	10.8 ± 0.1	10.3 ± 0.03	9.2 ± 0.1	6.0 ± 0.2*	8.1 ± 0.2*	9.2 ± 0.1*	9.0 ± 0.2*	5 .7 ± 0.2
TR2 - 300 µM[a]	11.0 ± 0.1	8.5 ± 0.5*	10.6 ± 0.1	9.6 ± 0.1*	5.5 ± 0.2*	4.4 ± 0.1*	7.6 ± 0.2*	10.2 ± 0.1*	10.3 ± 0.3	4 .9 ± 0.3*

Data are means ± SD of the last 3 days of each fermentation period. Means with an asterisk (*) are significantly different from control reactor (CR) within the same bacterial group, $P < 0.05$.
n.d., not detected
[a] 2,2'-dipyridyl concentrations in the feed medium of the reactors.
[b]one of three values below detection limit.
