# Supplementary material for: Iron Modulates Butyrate Production by a Child Gut Microbiota In Vitro
Source: mBio. 2015 Nov 17;6(6):e01453-15. doi: 10.1128/mBio.01453-15 (PMC4659462; doi:10.1128/mBio.01453-15)
Supplement: Table S2 — Primers used to enumerate specific bacterial groups and quantify gene expression by qPCR. [file mbo005152539st2.pdf]

Supplementary Table S2: Primers used to enumerate specific bacterial groups and quantify gene expression by qPCR

Primer	Sequence 5'-3'	Target	Source
Eub338F<br>Eub518R	ACTCCTACGGGAGGCAGCAG<br>ATTACCGCGGCTGCTGG	total 16S rRNA, *rrs* gene	(1)
Bac303F<br>Bfr-Femrev	GAAGGTCCCCCACATTG<br>CGCKACTTGGCTGGTTCAG	*Bacteroides* spp.	(2)
Firm934F<br>Firm1060R	GGAGYATGTGGTTTAATTCGAAGCA<br>AGCTGACGACAACCATGCAC	Firmicutes	(1)
Clep866mF<br>Clep1240mR	TTAACACAATAAGTWATCCACCTGG<br>ACCTTCCTCCGTTTTGTCAAC	*Clostridium* Cluster IV	(2)
RrecF<br>Rrec630mR	GCGGTRCGGCAAGTCTGA<br>CCTCCGACACTCTAGTMCGAC	*Roseburia* spp./*E. rectale*	(3)
Fprau223F<br>Fprau420R	GATGGCCTCGCGTCCGATTAG<br>CCGAAGACCTTCTTCCTCC	*Faecalibacterium prausnitzii*	(4)
EhalF<br>EhalR	GCGTAGGTGGCAGTGCAA<br>GCACCGRAGCCTATACGG	*E. hallii*	(2)
F_Lacto 05<br>R_Lacto 04	AGC AGT AGG GAA TCT TCC A<br>CGC CAC TGG TGT TCY TCC ATA TA	*Lactobacillus/Pedio-coccus/Leuconostoc* spp.	(3)
xfp-fw<br>xfp-rv	ATCTTCGGACCBGAYGAGAC<br>CGATVACGTGVACGAAGGAC	Bifidobacteria phosphoketolase gene	(5)
Eco1457F<br>Eco1652R	CATTGACGTTACCCGCAGAAGAAGC<br>CTCTACGAGACTCAAGCTTGC	*Enterobacteriaceae*	(4)
BCoATscrF<br>BCoATscrR	GCIGAICATTTCACITGGAAYWSITGGCAYATG<br>CCTGCCTTTGCAATRTCIACRAANGC	butyryl-CoA:acetate CoA-transferase gene, *butCoAT*	(6)
FeoB _F<br>FeoB _R	GAGCTGTTCGGCAAAACTTC<br>AGCAGTGCCACCAAAATACC	ferrous iron transport system gene in *R. intestinalis, feoB*	This study
ldh_ F<br>ldh_ R	TCGGAGATGGTCATGATTGA<br>TTCCGGCATCTACAATGTCA	L-lactate dehydrogenase gene in *R. intestinalis*, *ldh*	This study
pfl-AE_F<br>pfl-AE_R	TGGCGAAATTTTTCTGGAAC<br>TAGCCACAGCAGGGTAATCC	pyruvate-formate-lyase activating enzyme gene in *R. intestinalis,* pfl-AE gene	This study
pfo_F<br>pfo_R	TCCAGTATGACTCCAAGAA<br>TGGCAGTTGATCATGAA	pyruvate-ferredoxin-oxidoreductase gene in *R.intestinalis*, *pfo*	This study
hyd_ F<br>hyd_ R	ACTCATTCCAGGTCCGACAC<br>TAACTGGAAATCGGGCAATC	hydrogenase gene in *R. intestinalis*, *hyd*	This study

References

1. **Guo, X., X. Xia, R. Tang, J. Zhou, H. Zhao, and K. Wang.** 2008. Development of a real-time PCR method for Firmicutes and Bacteroidetes in faeces and its application to quantify intestinal population of obese and lean pigs. Lett Appl Microbiol **47:**367-73.
2. **Ramirez-Farias, C., K. Slezak, Z. Fuller, A. Duncan, G. Holtrop, and P. Louis.** 2009. Effect of inulin on the human gut microbiota: stimulation of *Bifidobacterium adolescentis* and *Faecalibacterium prausnitzii*. Br J Nutr **101:**541-50.
3. **Furet, J. P., O. Firmesse, M. Gourmelon, C. Bridonneau, J. Tap, S. Mondot, J. Dore, and G. Corthier.** 2009. Comparative assessment of human and farm animal faecal microbiota using real-time quantitative PCR. FEMS Microbiol Ecol **68:**351-62.
4. **Bartosch, S., E. J. Woodmansey, J. C. Paterson, M. E. McMurdo, and G. T. Macfarlane.** 2005. Microbiological effects of consuming a synbiotic containing *Bifidobacterium bifidum, Bifidobacterium lactis*, and oligofructose in elderly persons, determined by real-time polymerase chain reaction and counting of viable bacteria. Clin Infect Dis **40:**28-37.
5. **Cleusix, V., C. Lacroix, G. Dasen, M. Leo, and G. Le Blay.** 2010. Comparative study of a new quantitative real-time PCR targeting the xylulose-5-phosphate/fructose-6-phosphate phosphoketolase bifidobacterial gene (xfp) in faecal samples with two fluorescence *in situ* hybridization methods. J Appl Microbiol **108:**181-93.
6. **Louis, P., and H. J. Flint.** 2007. Development of a semiquantitative degenerate real-time pcr-based assay for estimation of numbers of butyryl-coenzyme A (CoA) CoA transferase genes in complex bacterial samples. Appl Environ Microbiol **73:**2009-12.
