# Supplementary material for: Iron Modulates Butyrate Production by a Child Gut Microbiota In Vitro
Source: mBio. 2015 Nov 17;6(6):e01453-15. doi: 10.1128/mBio.01453-15 (PMC4659462; doi:10.1128/mBio.01453-15)
Supplement: Text S1 — Supplemental materials and methods. Download [file mbo005152539s1.pdf]

Supplemental Material

Supplementary Materials and Methods

Fermentation medium design

The fermentation medium was similar, except the Fe content, to the previously described medium mimicking the chyme reaching the proximal colon of a child (1-4). Fe concentrations in fermentation medium were measured in duplicate by atomic absorption spectroscopy (SpectrAA-240K with GTA-120 Graphite Tube Atomizer Varion Techtron).

'Normal Fe' medium was formulated with 5.0 mg L^{-1} $FeSO_4$*$7H_2O$ and 50 mg L^{-1} hemin (Sigma-Aldrich) which resulted in an absolute Fe concentration in the fermentation medium of 8.2 ± 0.1 mg L^{-1} to approximate the daily Fe intake of a child as previously described (4).

Two levels of Fe deficiency were mimicked by preparing fermentation medium containing either 200 µM 2,2'-dipyridyl ('200 µM dip' medium), to generate moderate Fe deficiency, or 300 µM 2,2'-dipyridyl ('300 µM dip' medium), to generate strong Fe deficiency. Moreover, this low Fe media contained 0.1 mg L^{-1} hemin and no $FeSO_4$*H_2O and the absolute Fe concentration was 3.1 ± 0.3 mg L^{-1}.

Strong Fe supplementation was mimicked by adding 1.1 g L^{-1} $FeSO_4$*$7H_2O$ (217.8 mg Fe) to the fermentation medium ('High Fe' medium, absolute Fe concentration 245.4 ± 10.9 mg L^{-1}) containing 50 mg L^{-1} hemin. This resulted in a daily Fe supplementation of the reactor of 130.7 mg Fe with an inflow rate of 600 mL within 24 h. Although Fe supplementation in children should not exceed 60 mg per day (5, 6), we doubled the Fe concentration in the fermentation medium for the mechanistic objective.

qPCR procedures for microbiota analysis and gene expression

PCR reactions consisted of 2 x SYBR Green Mastermix (Life Technologies) or 2 x Kapa Sybr Fast qPCR Mastermix (Biolabo Scientifics Instruments, Switzerland) and 1 µL template genomic DNA in a total volume of 25 µL. Amplification consisted of an initial denaturation step at 95℃ for 10 min (20 s for Kapa Sybr Fast qPCR Mastermix) followed by 40 cycles of 95℃ for 15 s (3 s) and 60℃ for 1 min (30 s). A denaturation step was added to check for amplicon specificity. Samples were analyzed in duplicate and standard curves with the specific target 16S rRNA gene or functional gene were included in each run as previously described (4). Primers were used at a concentration of 0.2 µM for enumeration of bacterial groups and are listed in Supplementary Table S1. For butyryl-CoA:acetate CoA-transferase gene copies enumeration and gene expression analysis primer concentration was 1 µM and the qPCR protocol was adjusted as previously described (7).

RNA extraction from fermentation effluents and cDNA preparation

0.5 mL effluent samples were collected with a CO_2 flushed syringe directly from the reactors, mixed immediately with 1 mL RNAProtect for Bacteria (QIAGEN AG, Switzerland) and centrifuged at 5000 x g for 5 min. The pellet was shock-frozen in liquid nitrogen and stored until RNA extraction. For RNA extraction, pellets were thawed on ice, mixed with 1 mL cold TRI Reagent (Life Technologies) and 400 mg zirconium beads (0.1 mm) and cells were disrupted in a bead beater (4 x 40 sec, 5 m s^{-1}) with cooling on ice between cycles. After centrifugation (12000 x g, 10 min), supernatant was mixed with 200 µL ice cold chloroform (Sigma-Aldrich), centrifuged again as before and supernatants were subjected to RNA extraction with the High

Pure RNA Isolation Kit (Roche Diagnostics, Switzerland) according to the manufacturer's instructions. RNA quality was checked with an Agilent Bioanalyzer according to the manufacturer's instructions and RNA concentration was assessed with a Nanodrop® ND-1000 Spectrophotometer (Witec AG, Switzerland). 500 ng total RNA were transcribed to cDNA with the High Capacity cDNA Reverse Transcription Kit (Life Technologies) according to manufacturer's instructions.

Bacterial strain and culture conditions

Roseburia intestinalis L1-82 (DSM14610^T) was routinely cultured under strict anaerobic culture conditions at 37℃ in 10 mL yeast extract–casein hydrolysate–fatty acids (YCFA) medium (8) supplemented with glucose, starch and cellobiose (Sigma-Aldrich), each at a concentration of 2 g L^{-1}. Anaerobic culture methods were used for all experiments and YCFA medium was dispensed anaerobically in CO_2 flushed Hungate tubes closed with a butyryl-stopper before autoclaving (9).

Primer design and gene expression analysis for the R.intestinalis growth studies

CLC Genomic Workbench 4.9 (CLC Bio, Denmark) and Primer3Plus (10) were used to generate primers (Table 1) for the genes above based on the reference genomes of *R. intestinalis* L1-82 (DSM14610^T; NCBI accession PRJNA55267) and *R. intestinalis* XB6B4 (NCBI accession PRJNA197179). Expression of these genes including expression of the 16S rRNA gene (*rrs*) and butyryl-CoA:acetate CoA-transferase gene (*butCoAT*) was measured quantitatively with the primers described in Table 1 with a primer concentration of 0.2 μM or 1 μM for *butCoAT*. qPCR procedures were as described above. For standard curves, PCR amplicons for each

gene were generated with *R. intestinalis* DSM 14610^T genomic DNA, purified and DNA concentration was measured on a Nanodrop® ND-1000 Spectrophotometer (Witec AG) for calculation of copy numbers per µL. Serial dilutions were used as standard curve for the corresponding gene and included in each qPCR run to quantify gene expression.

References

1. **Macfarlane, G. T., S. Macfarlane, and G. R. Gibson.** 1998. Validation of a three-stage compound continuous culture system for investigating the effect of retention time on the ecology and metabolism of bacteria in the human colon. Microb Ecol **35:**180-7.
2. **Le Blay, G., J. Rytka, A. Zihler, and C. Lacroix.** 2009. New *in vitro* colonic fermentation model for *Salmonella* infection in the child gut. FEMS Microbiol Ecol **67:**198-207.
3. **Zihler, A., M. Gagnon, C. Chassard, A. Hegland, M. J. Stevens, C. P. Braegger, and C. Lacroix.** 2010. Unexpected consequences of administering bacteriocinogenic probiotic strains for *Salmonella* populations, revealed by an *in vitro* colonic model of the child gut. Microbiology **156:**3342-53.
4. **Dostal, A., S. Fehlbaum, C. Chassard, M. B. Zimmermann, and C. Lacroix.** 2013. Low iron availability in continuous *in vitro* colonic fermentations induces strong dysbiosis of the child gut microbial consortium and a decrease in main metabolites. FEMS Microbiol Ecol **83:**161-75.
5. **WHO.** 2002. Iron Deficiency Anemia; Assessment, Prevention, and Control; A guide for programme managers. Geneva, World Health Organization.
6. **CDC.** 1998. Centers for Disease Control and Prevention: Recommendations to Prevent and Control Iron Deficiency in the United States.
7. **Louis, P., and H. J. Flint.** 2007. Development of a semiquantitative degenerate real-time pcr-based assay for estimation of numbers of butyryl-coenzyme A (CoA) CoA transferase genes in complex bacterial samples. Appl Environ Microbiol **73:**2009-12.

8. **Pryde, S. E., S. H. Duncan, G. L. Hold, C. S. Stewart, and H. J. Flint.** 2002. The microbiology of butyrate formation in the human colon. FEMS Microbiol Lett **217:**133-9.

9. **Bryant, M. P.** 1972. Commentary on the Hungate technique for culture of anaerobic bacteria. Am J Clin Nutr **25:**1324-8.

10. **Untergasser, A., H. Nijveen, X. Rao, T. Bisseling, R. Geurts, and J. A. Leunissen.** 2007. Primer3Plus, an enhanced web interface to Primer3. Nucleic Acids Res **35:**W71-4.
